# Treatment of Glaucoma Patients with Flammer Syndrome

**DOI:** 10.3390/jcm10184227

**Published:** 2021-09-17

**Authors:** Katarzyna Konieczka, Josef Flammer

**Affiliations:** Department of Ophthalmology, University of Basel, 4056 Basel, Switzerland; josef.flammer@unibas.ch

**Keywords:** glaucoma, normal tension glaucoma, Flammer syndrome, calcium channel blockers, magnesium, nutrition, antioxidants

## Abstract

Flammer syndrome (FS) describes a phenotype characterized by the presence of primary vascular dysregulation along with a number of symptoms and signs. Although most people with FS are healthy, FS favors the occurrence of certain diseases, such as normal tension glaucoma. This is because disturbed autoregulation makes the eye more sensitive to intraocular pressure (IOP) spikes or blood pressure drops. Treatment of FS is generally appropriate when patients either suffer greatly from their symptoms or if we can assume that it has contributed to a disease. In glaucoma, this may be the case if the glaucoma damage progresses despite well-controlled IOP. Both the still sparse scientific studies and our long clinical experience suggest that FS-targeted therapy not only relieves the symptoms of FS but also slows the progression of glaucoma damage in selected cases. This description is intended not only to help affected patients but to also motivate clinicians and researchers to conduct therapy studies to confirm or refute our observations.

## 1. Introduction

Flammer syndrome (FS) [1,2,3,4,5] describes a phenotype characterized by the presence of primary vascular dysregulation [3,6] together with a combination of symptoms and signs that result from predisposition to generally increased sensitivity. The main focus is a modified, mostly increased response of the blood vessels to certain stimuli, such as cold or emotional stress, and the resulting phenomenon, such as cold hands. This combination of symptoms was first primarily observed in patients, especially those with normal tension glaucoma (NTG). Later, we noticed that the same combination of symptoms can also occur in healthy people but less frequently and usually less pronounced [1,3]. Therefore, we created a multiple-choice questionnaire that patients can fill out prior to consultation [5,7].

FS is not a disease but rather a predisposition that usually does not require treatment. However, treatment is recommended if diseases fostered by FS arise [7,8] or if people subjectively suffer from their symptoms. Although the syndrome is quite prevalent, there have only been a few studies dealing with the therapy. In contrast, there are many years of clinical experience. Therefore, the following recommendations are based on both studies and clinical experience. Although most people with FS are healthy and FS even seems to protect against atherosclerosis, it is a risk factor for some other diseases [8]. Here, we focus on glaucoma [3,6,7,9], specifically NTG [7].

Some recommendations concerning lifestyle management, nutrition, and drug therapy that have proven helpful in our clinical glaucoma practice are discussed here.

## 2. What Should Patients with FS Avoid?

Patients with FS generally observe themselves well, and they know what is good and what is not good for them. Nevertheless, they are usually grateful and relieved when their doctor discusses the following aspects.

### 2.1. Cold Exposure

Cold hands and/or feet are a leading symptom of FS [10]. That is why people with FS generally prefer to avoid the cold, and they often notice that they can fall asleep sooner if they warm up their feet or wear socks to bed. Others report symptoms such as chest pain when they drink cold liquids. While this is usually harmless, cold can also be dangerous in rare cases.

We were treating a FS patient with NTG who noticed a sudden increase in her visual field defect while skiing in very cold weather. The perimetry on the very next day revealed a new, large absolute scotoma.

Two female NTG patients independent of each other fell unconscious after jumping into the cold waters of the North Sea. Both ladies had to be rescued by their partners, and this happened twice to one of them. A young doctor with pronounced FS suffered a heart attack when he jumped into a cold swimming pool.

These clinical observations have also been confirmed by experimental studies [11,12]. The visual fields of glaucoma patients with FS temporarily worsened when they put one hand in cold water, while the visual fields of patients without FS remained stable [12].

### 2.2. Psychological Stress

Everybody experiences emotional stress from time to time, which can trigger a variety of different physical symptoms. In people with FS, these symptoms are mostly vascular. For instance, they notice cold hands or white and red spots on their face or neck [1,13].

Using thermographic images, we have observed that, in people with FS, some areas of the face cool down while other areas warm up at the same time during emotional stress.

A very successful young musician suffered from an NTG. During capillary microscopy of the nailfold, the cold provocation caused a blood flow standstill of 60 s. Later, when she told about her problems with her husband, the blood flow stopped for 130 s.

These symptoms and signs in the fingers are harmless. If, however, these phenomena occur in other organs, such as the eye, it can potentially cause damage.

One of our NTG patients with FS was stable for years, but within a three-month period, her visual field deteriorated very much. Only when we asked her specifically about stress did she describe heavy burden because of her daughter’s divorce.

Three independent bankers with FS had developed an anterior ischemic optic neuropathy (AION). Two of them became sick during the great financial crisis of 2008 and the third individual became so later but also after he lost money in the stock market.

Another young patient with FS had an argument with her superior. She developed an AION in her left eye on the same day and was treated in an emergency center with corticosteroids. They were ineffective, and she became blind in her affected left eye. Three months later, under similar conditions, she noticed visual disturbances in her right eye and visited our clinic. We diagnosed a fresh AION in her right eye and immediately initiated a full FS therapy (see below). Her visual field, visual acuity, and optic nerve head recovered. Today, many years later, this right eye is still healthy.

A man with FS in his mid-50s lost his job. On the same day on his way home, he caused a car accident. He was sent to a hospital with minor injuries. However, when he arrived at the hospital, he noticed that he could not see anything in one eye. An AION was diagnosed, but unfortunately, he was not transferred to us until a few days later. He remained blind in this eye.

An older teacher at a high school who had already lost one eye to an AION some years earlier had an argument with the parents of one of his pupils. That same evening, he developed an AION in his remaining good eye. Fortunately, we had the opportunity to start treatment the same evening, and his eye largely recovered. Today, many years later, the man is retired and is subjectively not disturbed by his visual field deficits and can read well with this eye. His FS symptoms have decreased.

A monk with FS was treated for an AION. A few weeks later, similar symptoms appeared in his other eye, and he finally came to us. We found a massive increase in retinal venous pressure (RVP), a phenomenon we often observe in FS patients [14,15]. After we decreased the RVP (see below), the first eye improved slightly and the second eye improved significantly.

Another man with FS and an advanced NTG felt significant pressure to perform in his job as a goldsmith. He took early retirement, and his FS symptoms subsided for the most part and the glaucoma stabilized.

A 14-year-old FS girl was under tremendous stress because she could not meet her school’s expectations. She developed an AION, and unfortunately, we did not see her until two weeks after the event. She remained blind in one eye, but her other eye has remained healthy for many years now with mild prophylactic therapy.

A young man with FS was responsible for the maintenance and repair of postbuses. One day, chips were introduced to acknowledge workers’ individual achievements. This put him under massive psychological and emotional pressure, and he developed a central serous chorioretinopathy [16,17]. An indocyanine green angiography revealed a distinct venous dysregulation in the choroid of the affected eye and, to a lesser extent, also in the unaffected eye. The patient completely recovered after he changed his job.

Even more frequently than AION, we have seen retinal vein occlusions after stress in otherwise completely healthy people with FS, including doctors in our hospital and professors from our university. A young sportswoman developed very high RVP up to a venous stasis retinopathy after breaking up with her boyfriend, but she recovered slowly once treatment was administered (see below). In another researcher, otherwise healthy, we also observed increased RVP when she realized she was going to lose her job at the university.

A student from a developing country was studying in Switzerland. He came under enormous stress because he was afraid that he would have to leave Switzerland before he could finish his studies. Three months after subjective visual disturbances, he was diagnosed with NTG with a new onset scotoma in the visual field.

Of course, stress simply cannot be avoided. However, it is possible to manage private and professional life in such a way that stress becomes less pronounced. There are also strategies, such as autogenic training and yoga, that can help a person deal with unavoidable stress. Sometimes professional support is needed, and if potentially dangerous vascular reactions persist, prophylactic drug therapy may make sense (see below).

### 2.3. Extreme Physical Activity

Exercise is healthy for everybody, including people with FS, as long as it is not too extreme. As we have observed that people with outdoor job suffer much less frequently from both FS symptoms and FS-associated diseases than people with indoor jobs [3,18], we recommend exercising and/or playing sports outdoors as often as possible. We have also observed that exposure to daylight reduces FS symptoms. With this in mind, we recommend exercising or playing sports outside during the day instead of inside or at night.

Occasionally, we have observed patients who exercise intensively for long periods without interruption, and some almost become addicted to it. Many FS patients jog or cycle very intensively. Reducing these extreme activities usually improves vascular regulation.

A young man with FS and advanced NTG rode his bike for several hours every day in a mountain area because he felt he needed it. While monitoring his blood pressure (BP) over a 24 h period, his systolic BP dropped to 65 mm Hg at night and sometimes dropped even lower. We recommended that he shorten his trips to about one hour a day. Fortunately, his nighttime BP dropped less and his glaucoma stabilized.

Patients with FS are also more sensitive to vibrations [3,4,18].

A young woman with FS noticed a slight earthquake, while other people in her environment did not notice it. Vibrations of the hands can lead to vasoconstrictions, and such individuals should not work with compressors. They are also more sensitive to mechanical traction. It is very common for FS patients to require more treatment and recovery time after whiplash injury.

### 2.4. Rapid Increase in Altitude

The response to lower oxygen concentrations at higher elevations is stronger in FS subjects than in individuals without FS, and they take longer to adapt to the condition.

A young lady with FS treated for a venous stasis retinopathy fell unconscious on a hot air balloon ride over the Alps. She regained consciousness immediately after the balloon dropped to a lower elevation.

A young FS man took a cable car from 1500 to 3200 m above sea level. He was unconscious when he arrived, so he was immediately taken back to the valley by gondola where he quickly recovered.

In a flight simulator study, we found that young healthy FS subjects were less able to tolerate reduced atmospheric pressure. For some of them, it was so bad that they had to abort the study, while subjects without FS tolerated it very well.

## 3. Nutritional Recommendations for Patients with FS

While most dietary advice is rightly aimed at reducing body weight, people with FS should make sure that their body mass index (BMI) does not fall too low. The lower a person’s BMI, the more intense the FS symptoms. A normal body weight should be aimed. Because fasting can exacerbate the symptoms of FS, we advise patients with FS against prolonged or intensive fasting [19].

Glaucoma patients with FS have increased oxidative stress [20,21], particularly in the mitochondria of neural axons. Food should therefore contain as many natural antioxidants, for example polyphenols, as possible. [22,23]. Please also refer to Section 4.2.9.

An increase in omega-3 fatty acids, especially in the form of seafood, can be helpful. It reduces FS symptoms by upregulating the uncoupling proteins, thereby increasing ATP- independent heat production.

Although the magnesium (Mg) plasma concentration in FS patients is usually normal, the diet should contain sufficient Mg. An Mg supplement is discussed below.

FS patients often have very low BP. While reduction of salt intake is generally rightly recommended, particularly for patients with arterial hypertension, FS patients with severe arterial hypotension should consume enough salt, especially in the evening, to avoid major drops in BP during sleep.

People with FS also have reduced feeling of thirst, which often leaves them dehydrated. Therefore, it is important that they make sure that they drink enough fluids, also in the evening before going to sleep.

## 4. Drug Treatment of Patients with FS

Of course, we only treat FS if necessary, and this is especially true for drug therapy. As ophthalmologists, we treat FS patients with eye diseases such as glaucoma (particularly NTG), retinal vein occlusions despite the lack of classical risk factors, and central serous chorioretinopathy. However, it is important to emphasize that FS does not only affect the eye [3,8,24]. Many of our patients or their relatives have also experienced nonocular diseases associated with FS, such as acute hearing loss, heart attacks despite lacking classical risk factors, certain autoimmune diseases, etc. [3,8]. The therapy that we employ is administered in a holistic manner, and we do not treat just one organ such as the eye.

In glaucoma, we primarily lower the intraocular pressure (IOP). This also makes sense for glaucoma patients with FS. FS patients usually have disturbed autoregulation and are therefore more sensitive to IOP peaks and BP dips. Often, however, IOP reduction and IOP stabilization alone are not sufficient. These are especially the cases in which we recommend treatment of FS.

### 4.1. Drug Sensitivity

Patients with FS often tell us that they cannot tolerate certain medications very well or apparently not at all in some cases and therefore prefer herbal remedies or homeopathic therapy. Based on our experience, however, these patients can actually tolerate these drugs if we prescribe much smaller doses than normal, which can be up to 10 times lower. Interestingly, the main effect usually remains, but the side effects disappear.

We draw the attention of patients with FS to the fact that their children are also more likely to have FS. The 18-year-old son of one of our NTG patients with FS was hospitalized at an internal medicine clinic due to general infection. During the stay, his BP dropped so low that he had to be given adrenaline intravenously. The resulting vasoconstrictive reaction was so violent that he lost fingers, toes, and the tip of his nose.

We recommend that FS patients start drug treatment at a very low dose whenever possible and then increase it slowly until they see the desired effect or until side effects occur. We recommend that a person with FS should be particularly cautious with all vasoconstrictive medications. Often, neither the doctors nor the patients notice that many different medications (e.g., some psychotropic drugs) have a vasoconstrictive side effect.

Based on our experience and also published information, surgery and especially anesthesia should be carefully planned for FS patients [25,26].

We investigated the effect of glaucoma drugs on corneal temperature. After one drop of brimonidine, the temperature of the cornea dropped for approximately 90 min due to the vasoconstrictive effect of brimonidine [27], whereas we observed no cooling under placebo. Interestingly, this effect was significantly stronger in patients with FS than in patients without FS (CTR NCT01201551, publication in preparation). Our glaucoma patients with FS generally did not tolerate brimonidine very well compared to patients without FS.

The symptoms of FS decrease significantly within a few years after menopause, and NTG thus usually (but not always) stabilizes. If women take postmenopausal hormone therapy that contains estrogen, both the FS symptoms and the glaucoma can worsen again. In case of doubt, the regulation of the eye blood circulation can be measured before and after the beginning of therapy.

### 4.2. Pharmaceutical Improvement of Regulation of the Microcirculation

Primary vascular dysregulation [3,6], the core component of FS, has not been known for very long. It is therefore not surprising that there are only a few types of drugs available, and the number of clinical studies is limited. Unfortunately, the pharmaceutical industry has hardly addressed this issue until now. Nevertheless, we are already able to effectively help these patients. We have had good experience with calcium channel blockers and magnesium.

#### 4.2.1. Calcium Channel Blockers (CCBs)

In ex vivo studies, we have demonstrated that CCBs significantly reduce the effect of endothelin-1 (ET) in ocular circulation [28,29]. In FS patients, we found a slight increase in plasma levels of ET [30]. However, even more important was our observation that the lower the BP, the greater the ET sensitivity in these patients [31].

Even before we knew this rational justification for CCB treatment, we already had clinical experience with it [5]. The positive visual field response to CCBs in certain patients was one of the cornerstones of discovering primary vascular dysregulation and FS. We have conducted various visual field studies. Over time, we noticed that it was always the same patients who showed a visual field response, whether it was an improvement under caroboanhydrase inhibitors [32], CCBs [33], and CO_2_ respiration [34] or a worsening caused by cold provocation [12]. Moreover, the visual field response occurred in the same patients who also showed prolonged arrest in blood circulation of the nailfold after cold provocation and shortening of this arrest after CCBs. Controversial discussions regarding the benefits associated with CCBs began to surface, particularly for glaucoma patients [35]. We had to clear up a lot of misunderstandings. First of all, we cannot simply expect a positive outcome in all patients; treatment only works if primary vascular dysregulation or FS is actually present. There were also fears of a "steel effect", which means that the vessels in healthy areas would be dilated, so even less blood would flow in the diseased areas. However, this is unlikely as such a steel effect would hardly explain visual field improvements, and it became evident that CCBs dilate pathologically contracted blood vessels more than healthy ones.

Others feared that CCBs would lower the BP of such patients even further and that this would be dangerous, particularly for glaucoma patients. We address this risk based on the following considerations: (a) we use very low doses that hardly ever lower BP; (b) the BP lowering effect of CCB is either small or nonexistent in patients who already have low BP; (c) animal experiments have shown that nifedipine increases ocular blood flow, even when it reduces BP [36]; and (d) if the ocular perfusion would decrease, we would not observe stabilization or even improvement but rather a deterioration of the visual field.

Others have assumed that fat-soluble (centrally acting) CCBs (e.g., nimodipine) would be better than water-soluble (peripherally acting) CCBs, such as nifedipine. This applies to diseases of the brain and retina as long as the blood-brain or blood-retina barrier is intact. For glaucoma, however, we have had the opposite experience, which may be explained by the fact that there is actually no blood-brain barrier in the optic nerve head [23]. It is important to note that studies comparing different CCBs in glaucoma patients are yet to be undertaken. In very severe cases, such as acute AION in FS patients, we start with a combination of nifedipine with nimodipine and then stop the nifedipine after a few days.

Under normal condition, we start with 1 mg nifedipine (i.e., 1 drop of a nifedipine solution) orally per day and then slowly increase the dose to 2, 3, or more mg depending on the patient, the BP, and the disease we are targeting. As nifedipine has a short half-life, patients dilute it in a liquid of their choice and drink it throughout the day. Because nifedipine is sensitive to light, we recommend using a light-protected bottle or keeping it in the dark (e.g., in the refrigerator or cabinet).

Fortunately, the half-life of the effect is significantly longer than the half-life of the blood level. Intake during the day instead of at night is desirable as thermographic studies have revealed that FS patients have vascular dysregulation during the day but not when they are asleep.

Many doctors hardly believe that such low doses could have an effect. Let us illustrate this by example. A researcher working for a pharmaceutical company in Basel told us about the fate of her father who lived in a developing country. He was diagnosed with NTG, and his ophthalmologist noticed a fast progression since he was on dialysis. In addition, the patient noticed a temporary deterioration in his vision after dialysis. Unfortunately, it was not possible for him to travel to us for an examination. We have observed similar events in patients on dialysis. They all had increased ET levels in the blood, which resulted in increased RVP, which contributed to progression of glaucoma damage. Assuming a similar situation, we recommended a therapy with a relatively low dose of nifedipine. A few weeks later, we received a letter from the patient informing us that his vision had improved and that his visual field deficits had decreased after the initiation of 1 and then 2 mg of nifedipine per day.

If a higher dose is necessary or desired, we replace nifedipine with amlodipine (5 mg once a day). Amlodipine has effects that are similar to nifedipine, but it has a longer half-life. However, unfortunately, it is only available in doses of 5 mg or higher.

Although FS patients usually have rather low BP, some of them develop high BP in old age. In such cases, we recommend an antihypertensive treatment containing a low dose of a CCB (e.g., a combination of an ACE inhibitor with 5 mg amlodipine).

We know that ET increases RVP [37], but not every high RVP is ET induced. Accordingly, one cannot lower every RVP with nifedipine. In addition, CCBs, and thus nifedipine, only inhibit ET-induced influx of calcium from the outside into the cell but not ET-induced release of calcium from the cell’s internal storage. This means that we can reduce but not completely eliminate the effect of ET. In addition, the venous resistance that leads to the increase in RVP does not always occur at the level of the optic disc. This can also be further in the retina or deeper in the optic nerve. In such cases, CCBs that are less water soluble, such as nimodipine, help better.

CCBs have the well-known effect of lowering blood pressure and side effects such as flush or ankle edema. However, as we prescribe extremely low doses (much lower than usually prescribed by internists), we see such side effects extremely rarely. Nevertheless, to be on the safe side, we recommend 24 h blood measurement before and after starting therapy with low-dose CCB.

#### 4.2.2. Endothelin Blockers

Endothelin blockers are particularly interesting [38]. Unfortunately, the benefit for FS subjects has not yet been investigated in detail, and they are not yet approved for this application. However, we know that RVP is mainly regulated by ET and that ET blockers reduce increased RVP [39]. Endothelin traps and the delivery of artificial transcription factors are also under investigation [40].

#### 4.2.3. Magnesium

Magnesium (Mg) is a physiological CCB. We have shown that Mg reduces the effect of ET both in vitro and in ex vivo [41]. We have further observed a slight improvement in the visual field of glaucoma patients with FS [42]. Mg has only mild side effects, such as diarrhea, which disappears after reducing the dose. Often, it is better tolerated by taking it together with yoghurt. We normally use 10–20 mmol of Mg per day, but there are only a few studies in the available literature and the effect is relatively small. We normally start treatment with Mg, and if the effect is insufficient, we combine it with a low dose of CCB.

#### 4.2.4. Betaxolol

Studies have demonstrated that glaucoma patients treated with betaxolol have a smaller rate of visual field deterioration than patients treated with timolol despite the fact that betaxolol reduces IOP less than timolol [43]. This can be explained by a slight calcium channel blocking effect of betaxolol. As betaxolol is beta-1 specific, it is generally tolerated by most (but not all) FS patients.

#### 4.2.5. Triflusal

Triflusal is a compound related to aspirin. However, in contrast to aspirin, it leaves the arachidonic acid pathway intact, favors the production of nitric oxide (NO), and increases the concentration of cyclic nucleotide in endothelial cells, which results in peripheral vasodilatation [44]. We do not have much experience with it because it is not yet on the market in Switzerland.

#### 4.2.6. Propranolol

Propranolol is a beta-blocker as well as a weak CCB. Taking very low doses (5–10 mg per day) for days or weeks help some FS patients when emotional stress is unavoidable and causes symptoms.

#### 4.2.7. Carbonic Anhydrase Inhibitors (CAI)

Acetazolamide is used to reduce both IOP and intracranial pressure. It also dilatates eye and brain vessels [45] and is therefore also used to study the cerebral perfusion reserve. Many decades ago, we and others found that acetazolamide can improve the visual fields in certain glaucoma patients [46]. Later, we realized that these patients had FS. With acetazolamide, we can determine whether some of the visual field defects are still reversible. Acetazolamide is rarely used as a long-term therapy because of its side effects.

CAIs used locally, such as dorzolamides, have fewer systematic side effects and also improve blood flow, albeit to a lesser extent than acetazolamides [47]. Therefore, they are ideal for glaucoma patients with FS despite their limited ability to lower IOP.

#### 4.2.8. NO Donators

NO donators are theoretically interesting for treatment of FS. Unfortunately, very little research has been done on the use of such drugs in FS patients. Nevertheless, it has already been shown that nitrates can lower RVP (ARVO Annual Meeting Abstract, 2017).

Calcium-L-methylfolate (the biologically active form of folic acid) not only reduces homocysteine but also increases NO production via activation of the NO synthetase, thereby improving vasodilatation. Vitamin supplementation containing L-methylfolate (Ocufolin^®^ forte) has shown promise. It improves diabetic and hypertensive retinopathy [48], conjunctival microcirculation [49], and retinal blood flow in diabetes patients [50]. Ocufolin^®^ forte has been studied little in glaucoma so far. However, we already know that it reduces elevated retinal venous pressure (in preparation).

#### 4.2.9. Antioxidants

We have already emphasized the importance of oxidative stress and antioxidant nutrition in the nutritional recommendations section. Although oxidative stress can occur systemically, local stress in certain organs and cells is even more important. Even within a cell, oxidative stress is often very localized (e.g., in mitochondria). Therefore, we cannot simply reduce all oxidative stress with any antioxidant. For example, in patients with glaucoma, stress occurs mainly (but not only) in the mitochondria of the axons in the optic nerve head. A balanced varied antioxidative diet contains molecules that reach these sites. The situation is different with antioxidative supplementation or therapy. Here, one must be purposefully selective. Ginkgo biloba, for example, has proven to reach the mitochondria of axons and exert their effect there. Other molecules are also promising and are currently being investigated (e.g., vitamin supplementation containing calcium-L-methylfolate). Doses of any antioxidants that are too high should imperatively be avoided because all antioxidants become prooxidants if the concentration in the body is too high.

### 4.3. Pharmaceutical Treatment of Systemic Hypotension

Arterial hypertension (high BP) is a frequent and well-known risk factor for many diseases. Correspondingly, there are also many treatment options. Less well known, however, is the fact that arterial hypotension (low BP), one of the leading FS symptoms [51,52], can also be a risk factor. Many studies have shown that low BP, increased BP fluctuations, nocturnal dips of BP, and orthostatic hypotension increase the risk of occurrence and progression of glaucomatous damage [53]. We are therefore often asked by patients what they can do to increase BP.

We always start with simple interventions, such as increasing salt (sodium chloride) intake mostly in the evening and physical activity. To better control the intake of additional salt, the pharmacist can prepare salt tablets. If the salt is poorly tolerated, it can be taken together with tomato juice.

FS patients should avoid certain drugs as much as possible. Many medications, especially sleeping pills and sedatives, have a side effect of lowering BP. The advantages and disadvantages of such treatments must therefore be weighed against each other.

In the case of orthostatic hypotension, we recommend that patients get up slowly in the morning. We recommend support stockings for people who are in a profession that requires them to stand (or sit) for a long time.

If all this is not sufficient, pharmacological therapy may be considered in rare cases. Although vasoconstrictive drugs are relatively often prescribed for this purpose, they reduce blood flow to the eyes despite an increase in BP and are therefore counterproductive in such cases.

We have had good experiences with a very low dose of fludrocortisone (2 × 0.1 mg per week) [54]. Fludrocortisone is a mineralocorticoid and not a glucocorticoid and therefore has fewer side effects than glucocorticoids.

The good news is that BP does not necessarily need to be totally normalized. In patients with severe hypotension, even a slight increase in BP leads to significantly better vascular regulation.

## 5. What Does This All Mean in Practice?

We already mentioned that not all people with FS need treatment. The good news is that very different FS-related phenomena usually respond to treatment in parallel. We found that FS patients often display the following disease signs at the same time: NTG [7], disturbed autoregulation of ocular blood flow [55], increased retinal venous pressure [14], and optic nerve compartment syndrome [3,56]. In most cases, treatment with a low dose of nifedipine (mostly combined with magnesium) simultaneously improved the regulation of retinal vessels, reduced RVP [57], and reduced optic nerve compartment syndrome [56].

How do we ensure that a chosen therapy works? In the end, the main criterion is organ and system function (e.g., the visual field in glaucoma). However, in most cases, we would like to see the effect more quickly. There are subjective criteria. If, for example, a patient notices that his hands have become warmer, then we are very likely on the right track.

However, there are also helpful objective parameters. A reduction of the RVP or an improved reaction of the retinal vessels to flickering light or a shortening of the flow standstill after cold provocation in nailfold capillary microscopy shows us that the treatment has a positive effect.

## 6. Conclusions

If glaucoma damage progresses despite normal or normalized IOP, then vascular factors are usually involved [6]. One of the vascular factors is primary vascular dysregulation [3], the main component of FS. There are several reasons why FS may be a risk factor for glaucoma damage. Patients with FS are more likely to have (a) impaired autoregulation [55], (b) low blood pressure [4,51], (c) high retinal venous pressure [14], and (d) activated astrocytes [58].

Therefore, we can expect that treatment of FS will improve the prognosis of glaucoma. However, this is yet to be proven in controlled long-term studies.

## Data Availability

Not applicable.
